# Multi spectroscopic investigations with molecular docking and molecular dynamics simulation of the binding mechanism of molnupiravir to bovine serum albumin

**DOI:** 10.1186/s13065-025-01645-5

**Published:** 2025-10-27

**Authors:** Fotouh R. Mansour, Nada Elhosary, Samar H. Elagamy, Aya Barseem, Amira H. Kamal

**Affiliations:** 1 Department of Pharmaceutical Analytical Chemistry, Faculty of Pharmacy, Tanta University, Elgeish Street, The Medical Campus of Tanta University, Tanta, 31111 Egypt; 2 Department of Medicinal Chemistry, Faculty of Pharmacy, King Salman International University (KSIU), South Sinai, Egypt; 3 Pharmaceutical Analysis Department, Faculty of Pharmacy, Menoufia University, Menoufia, Egypt; 4 Department of Pharmaceutical Analytical Chemistry, Faculty of Pharmacy, Menoufia National University, Km Cairo-Alexandria Agriculture Road, Menofia, Egypt

**Keywords:** Molnupiravir, COVID-19, Bovine serum albumin, Fluorescence, Synchronous emission

## Abstract

Molnupiravir (MOL) is a recently developed oral antiviral drug. MOL has been clinically evaluated for its efficacy against COVID-19. In this work, we utilize UV-absorption spectroscopy, fluorescence spectroscopy, synchronous fluorescence, molecular docking and molecular dynamic (MD) simulation to study the interaction between MOL and bovine serum albumin (BSA). The fluorescence spectroscopic analysis indicated that MOL effectively quenched the intrinsic fluorescence of BSA. To elucidate the fluorescence quenching mechanism, the interaction of MOL with BSA was examined at five distinct temperatures (285 K, 290 K, 295 K, 303 K and 308 K). The quenching constants diminished as the temperature increased, suggesting a static quenching mechanism, as evidenced by the Stern–Volmer plots of F0/F against the molar concentrations of MOL. This was further confirmed by UV absorption spectroscopy. Thermodynamic analysis revealed a 1:1 stoichiometry for MOL binding to BSA, with a negative Gibbs free energy change (ΔG), confirming the spontaneous nature of binding. Competitive binding experiments using site-specific markers as well as molecular docking studies showed that MOL binds to site II. Synchronous fluorescence spectroscopy indicated that MOL was binding around tyrosine (Tyr) residues of the protein. These findings were further confirmed by the molecular docking and MD simulation studies. This research could provide valuable insights into the pharmacokinetics and pharmacodynamics of MOL, which could contribute to the development of more effective antiviral drugs.

**Graphical Abstract:**

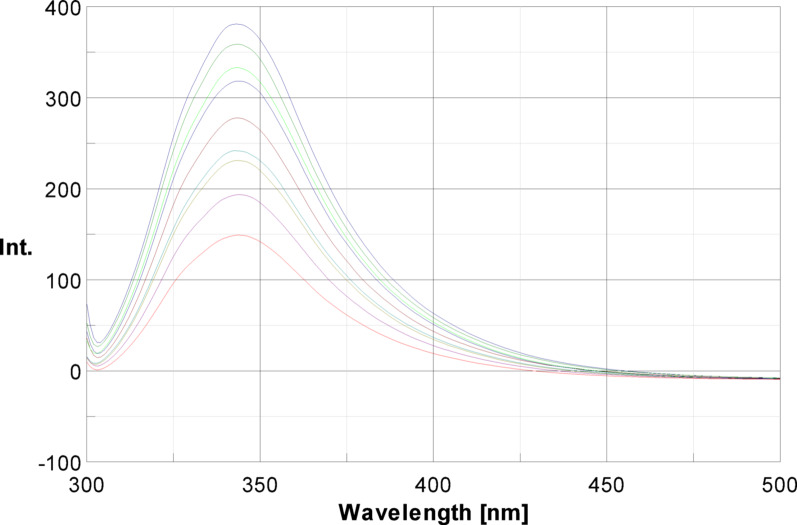

**Supplementary Information:**

The online version contains supplementary material available at 10.1186/s13065-025-01645-5.

## Introduction

**Fig. 1:**
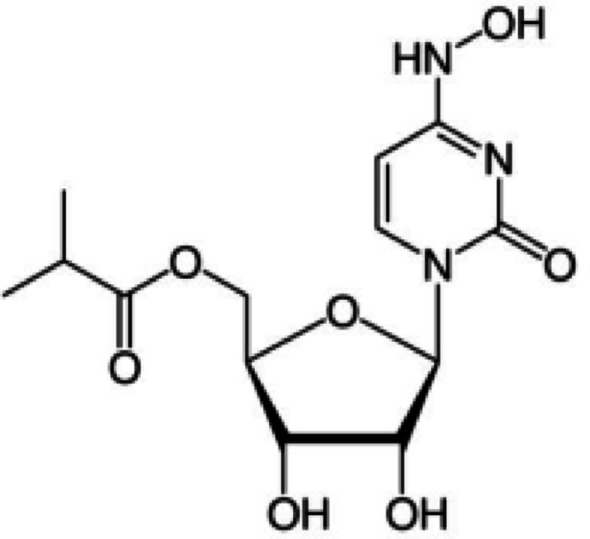
The chemical structure of Molnupiravir

Molnupiravir MOL is a pyrimidine ribonucleoside analog having a chemical structure shown in Fig. 1. MOL is the first oral antiviral medication discovered by Drug Innovation Ventures at Emory (DRIVE). In November 2021, MOL received its initial approval in the United Kingdom for the management of mild to moderate COVID-19 in adults with a confirmed positive SARS-CoV-2 medical test and at least one risk factor that makes them more vulnerable to serious illness. This newly approved antiviral has demonstrated substantial efficacy in reducing hospitalization and fatality rates in mild COVID-19 cases, making it a potentially promising tool against SARS-CoV-2 [1–3]. The majority of individuals affected by Corona virus disease 2019 experience a clinical progression that can lead to severe illness necessitating hospitalization. During the replication process, MOL, a molecule of the nucleoside analog N4-hydroxycytidine, inserts errors into the viral RNA, leading to an accumulation of mutations and ultimately inhibiting the virus’s ability to replicate and spread [4, 5].

 Serum albumin is a protein which was studied widely. It enhances the transportation of both exogenous and endogenous substances to their targets. In order to formulate novel medications and modify dosages of already-approved medications to achieve the desired therapeutic effects, the intensity of the interaction concerning medications/ligands and serum albumin is essential [6, 7].

The binding interaction concerning pharmaceuticals with serum albumin is physiologically critical for the transport, distribution, and metabolic processing of drugs within biological systems. Investigating the binding interactions concerning drugs and serum albumin enhances our comprehension of the mechanisms of action and metabolic pathways of these drugs. In general, weak protein binding tends to result in a short-term action or limited distribution, whereas strong binding typically results in a reduction of drug concentration, as pharmacological activity depends on the unbound fraction of the drug [8].

Drug-serum albumin binding interactions have been widely evaluated using BSA due to its affordability, availability, and structural resemblance to human serum albumin (76%). The three domains (I, II, and III) forming up the amino acid chain of BSA are further subdivided into two sub-domains (A and B), which are further subdivided by disulfide bonds into nine loops [9, 10].

There are many analytical methods reported for determining of MOL [11–13] such as RP-HPLC method [14], LC-MS/MS methods applied to biological fluids [15–18]. Other methods, including method using carbon quantum dot nano probes [19], and spectrophotometric method using silver-nanoparticles have also been found [20].

This study aims to provide a comprehensive analysis of the protein binding interaction between MOL and BSA using multiple spectroscopic techniques along with thermodynamic analysis, molecular docking and dynamic simulation studies. Although previous literature has explored similar interactions [21–24], the novelty of this work particularly when compared to Begum et al. [24] is that their study was limited to spectroscopic analyses, whereas this study combined molecular docking with triplicate 100 ns molecular dynamics simulations, supported by thermodynamic profiling and site marker competition assays. This integrative approach provides a deeper mechanistic understanding of the MOL–BSA binding. The research conducted could provide valuable insights into the pharmacokinetics and pharmacodynamics of MOL, potentially contributing to the development of more effective antiviral drugs [25]. This work also aids in a better understanding of the interaction between MOL and MOL-related structures with BSA.

## Experimental

### Materials and reagents

BSA (certified purity > 98.50%) was obtained from BIOMARK laboratories (Pune, Maharashtra, India). Molnupiravir (purity 99.90%) was kindly supplied by Global Napi Pharmaceutical Industries (Giza, Egypt). Sodium chloride (purity > 99%) was purchased from Acros Organics (Geel, Belgium). Tris(hydroxymethyl) aminomethane hydrochloride (Tris-HCl) (certified purity 99.90%) was purchased from Sigma Aldrich (St. Louis, Missouri, United States). Double-distilled water was used in this research.

### Instrumentation

A Jasco spectrofluorometer FP-6300 (Tokyo, Japan) with a 5 nm slit width and a 1000 nm/min scanning speed was utilized for the fluorescence analyses. Spectra Manager Software version 1.55 was used to run the instrument. UV-1800 PC double-beam Shimadzu spectrophotometer (Kyoto, Japan), 1 cm quartz cells and UV Probe software were employed to perform ultraviolet measurements. With a scanning speed of 400 nm/sec, zero-order UV spectra in the 200–400 nm region were obtained at 0.1 nm sampling intervals. Using Tensor27-S2887 FT-IR spectrometer (Bruker, Germany) with a DTGS detector, Fourier-transform infrared analysis was carried out for observations between 4000 and 1000 cm^−1^. For the experiments, a pH meter from HANNA (USA) and a digital balance made by Sartorius (Germany) were used.

### Preparation of reagents and solutions

A Tris-buffer solution (0.05 M) was obtained by dissolving 0.61 g of Tris base and 0.8766 g of NaCl (0.15 M) in 100 mL of water, then adjusting pH to 7.4 using conc. HCl. A BSA stock solution with a concentration of 50 µM was freshly obtained by dissolving 0.166 g of BSA in 50 mL of Tris-HCl buffer. The MOL stock solution (1 mM) was obtained by dissolving 16.46 mg of MOL in 50 mL of water. Different aliquots (0.6–1.8 mL) of the MOL stock solution were transferred to a series of 10-mL volumetric flasks to prepare fresh sample solutions. Each flask contained 1 mL of the BSA stock solution (50 µM), and the volume was adjusted to 10 mL using the Tris-HCl buffer solution.

### Methodology

#### UV-Spectrophotometry

UV absorption spectra of BSA (5 µM) were examined at wavelengths between 250 and 330 nm with different MOL concentrations (60, 120, and 180 µM) at pH 7.4. The experimental measurements were performed against a blank containing all corresponding concentrations of MOL. A temperature of 295 K was used for the experiments using Tris-HCl buffer as a solvent.

#### FT-IR spectroscopy

FT-IR spectra of 3 µM bovine serum albumin solution in tris-HCl buffer, BSA (3 µM) in the presence of MOL (180 µM), and MOL alone (180 µM) at pH 7.4 were recorded using Tensor27—S2887 FT-IR spectrometer (Bruker, Germany) from 4000 to 1000 cm^− 1^. Baseline correction and water vapor subtraction were applied prior to analysis.

#### Fluorescence spectroscopy

The fluorescence of the tyrosine (Tyr) and tryptophan (Trp) residues observed for solutions that have a fixed BSA concentration 3 µM and different concentrations of MOL (30–180 µM) at pH 7.4. The emission spectra were obtained between 300 and 500 nm. The excitation wavelength was 295 nm, at temperatures 285, 290, 295, 303, and 308 K. Corrections for the inner filter effect were considered unnecessary in our study, as the combined absorbance of the BSA-MOL mixture at the highest tested concentrations remained below 0.05 at both the excitation and emission wavelengths.

Initially, the synchronous fluorescence spectra of BSA were acquired at a concentration of 3µM, and then its mixtures with MOL ranged from 30 to 180 µM at pH 7.4. These spectra were obtained at spectral intervals of Δλ = 15 nm as well as Δλ = 60 nm, with a 10 mm path quartz cell and scanning rate of 1000 nanometers per minute (nm/min).

#### Study of the preferential binding site of MOL on BSA

This study was conducted using two specific site probes: indomethacin for site I and diazepam for site II. In these experiments, the concentrations of BSA and the site probes were kept constant at 3 µM and 10 µM, respectively, while the concentration of MOL was varied from 30 to 180 µM. Fluorescence intensity was measured at 350 nm with excitation at 295 nm, and all measurements were carried out at 295 K and pH 7.4. The binding equilibrium constants (Ka) of MOL to BSA alone, indomethacin-BSA, and diazepam-BSA complexes were compared.

### Molecular docking

Molecular docking simulations were conducted using AutoDock4 (v4.2.6) [26] to elucidate the binding interaction between MOL and Bovine Serum Albumin (BSA). The crystal structure of BSA was obtained from the Protein Data Bank (PDB ID: 4OR0) [27]. Water molecules and bound ligands were removed, and polar hydrogens and Kollman charges were added using AutoDock Tools (ADT v1.5.7). The structure was then saved in PDBQT format.

ChemBioDrawUltra was utilized to create the structure of the MOL (keto-oxime tautomer), that is the most energetically stable and energy-minimized using the MMFF94 force field in Avogadro. Gasteiger charges were assigned, and torsional flexibility was set in ADT. The ligand was also saved in PDBQT format.

A grid box was defined to encompass Site IIA and Site IIIA of BSA. The grid box dimensions were set to 60 × 60 × 60 Å with a spacing of 0.375 Å, and the grid was centered around the coordinates of subdomain IIIA based on literature precedents. Docking was performed using the Lamarckian Genetic Algorithm (LGA) with 100 runs and 2,500,000 energy evaluations.

The best binding pose was selected based on the lowest binding energy and the visual inspection of interactions. Molecular visualization and interaction analysis were performed using Discovery Studio Visualizer (version 20.1.0.19295) developed by Dassault Systemes BIOVIA Corporation.

### Molecular dynamics simulation

To investigate the dynamic stability and interaction behavior of the BSA–MOL complex, MD simulations were performed using GROMACS 2021.5. The crystal structure of BSA (PDB ID: 4OR0) was prepared by removing all heteroatoms and crystallographic waters, followed by hydrogen addition and solvation in a cubic box using the TIP3P water model. Sodium counter ions were added to neutralize the system.

The ligand MOL was parameterized using the General AMBER Force Field (GAFF), with AM1-BCC partial charges assigned via ACPYPE. The system underwent energy minimization using the steepest descent algorithm, followed by equilibration under constant volume (NVT, 100 ps) and constant pressure (NPT, 100 ps) conditions. The temperature and pressure were maintained at 300 K and 1 bar using the velocity-rescale thermostat and the Parrinello–Rahman barostat, respectively.

To ensure statistical robustness and reproducibility, the MD simulations were conducted in triplicate. Each run was 100 ns in length and initiated with different random velocity seeds. Trajectory analyses were performed using GROMACS built-in tools to compute root-mean-square deviation (RMSD), root-mean-square fluctuation (RMSF), hydrogen bonding, and radius of gyration (Rg).

## Results and discussion

BSA was selected as the model protein for this study because of its high structural and sequence homology with human serum albumin (HSA), as well as its widespread use in protein–ligand binding research. BSA is inexpensive, readily available, and provides a well-established platform for probing drug–albumin interactions under controlled laboratory conditions. Although HSA is more physiologically relevant for clinical translation, results obtained with BSA are generally considered predictive due to their similar binding domains and functional properties. Therefore, BSA was used here as a representative model to elucidate the interaction of MOL with serum albumin.

### Study of fluorescence quenching of bovine serum albumin by molnupiravir

**Fig. 2:**
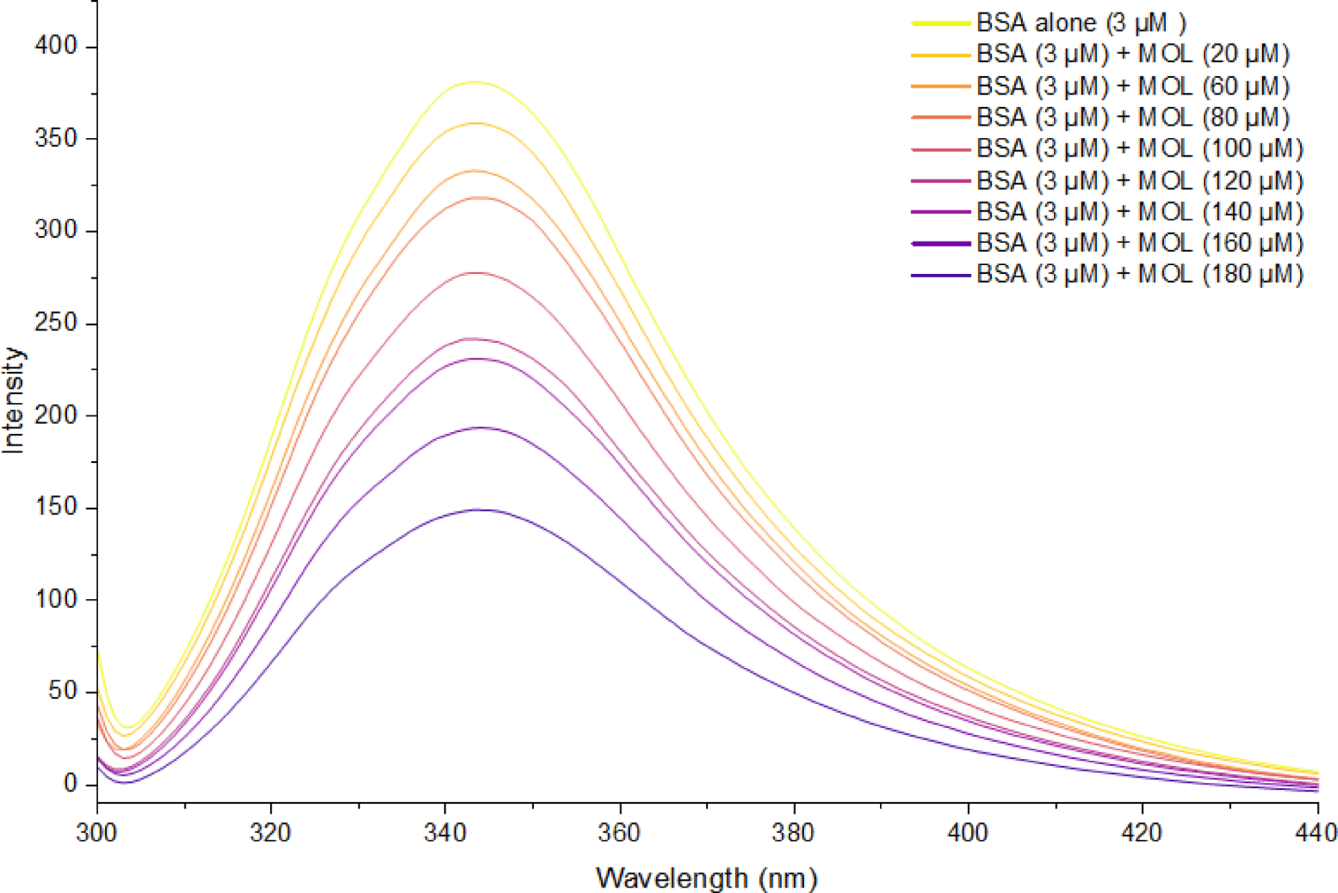
Fluorescence quenching spectra of BSA 3 µM with varying concentration of Molnupiravir 0–180 µM, at 290 K, pH = 7.4, with an excitation wavelength of 295 nm

Fluorescence techniques are crucial for studying ligand-protein interactions due to their sensitive, versatile, and non-destructive nature, allowing investigation into the structure, dynamics, and interactions of proteins [28]. The intrinsic fluorescence emitted by the protein exhibits remarkable sensitivity to minor changes in protein conformation or environmental conditions caused by interactions with ligand [29]. In this study, the excitation wavelength was set at 295 nm then the intensity of BSA fluorescence measured at 348 nm. The fluorescence intensity was found to be decreased upon the addition of different concentrations of MOL. The observed quenching phenomenon exhibited a direct correlation with the concentration of MOL (Fig. 2).

Fluorescence quenching can occur through several mechanisms, with static and dynamic processes being two primary types. In static quenching, a complex forms between the fluorophore and quencher, resulting in quenching. Conversely, dynamic quenching involves collisions between the quencher and fluorophore molecules. These mechanisms can be well-known by examining the effect of temperature on the quenching rate constant [30]. In static quenching, higher temperatures typically destabilize the quencher-fluorophore complex, leading to a decrease in the quenching rate constant. In dynamic quenching, however, increased temperatures enhance molecular diffusion and collisions between the quencher and fluorophore, thereby raising the quenching rate constant [31]. There was an inner filter effect due to the overlap presents between the MOL UV absorption spectrum and the excitation wavelength of BSA (Fig. S1). To explore other mechanisms, the fluorescence quenching was investigated in this study at temperatures of 290 and 295 as well as 303 K.

Utilizing the Stern–Volmer equation, the quenching mechanism was identified (Eq. 1):

1 $$\frac{{\text{Fo}}}{\text{F}}=1+{\text{K}_{\text{sv}}}\left[ \text{Q} \right]$$

The fluorescence intensities of BSA in absence of MOL are indicated as ${\text{Fo}}$ while those measured in the presence of MOL are referred to as ${\text{F}}$. The term ${\text{{K}}}_{\text{{sv}}}$ represents the Stern–Volmer quenching rate constant. MOL concentration represents the quencher concentration $\left[{\text{Q}}\right]$.

**Fig. 3:**
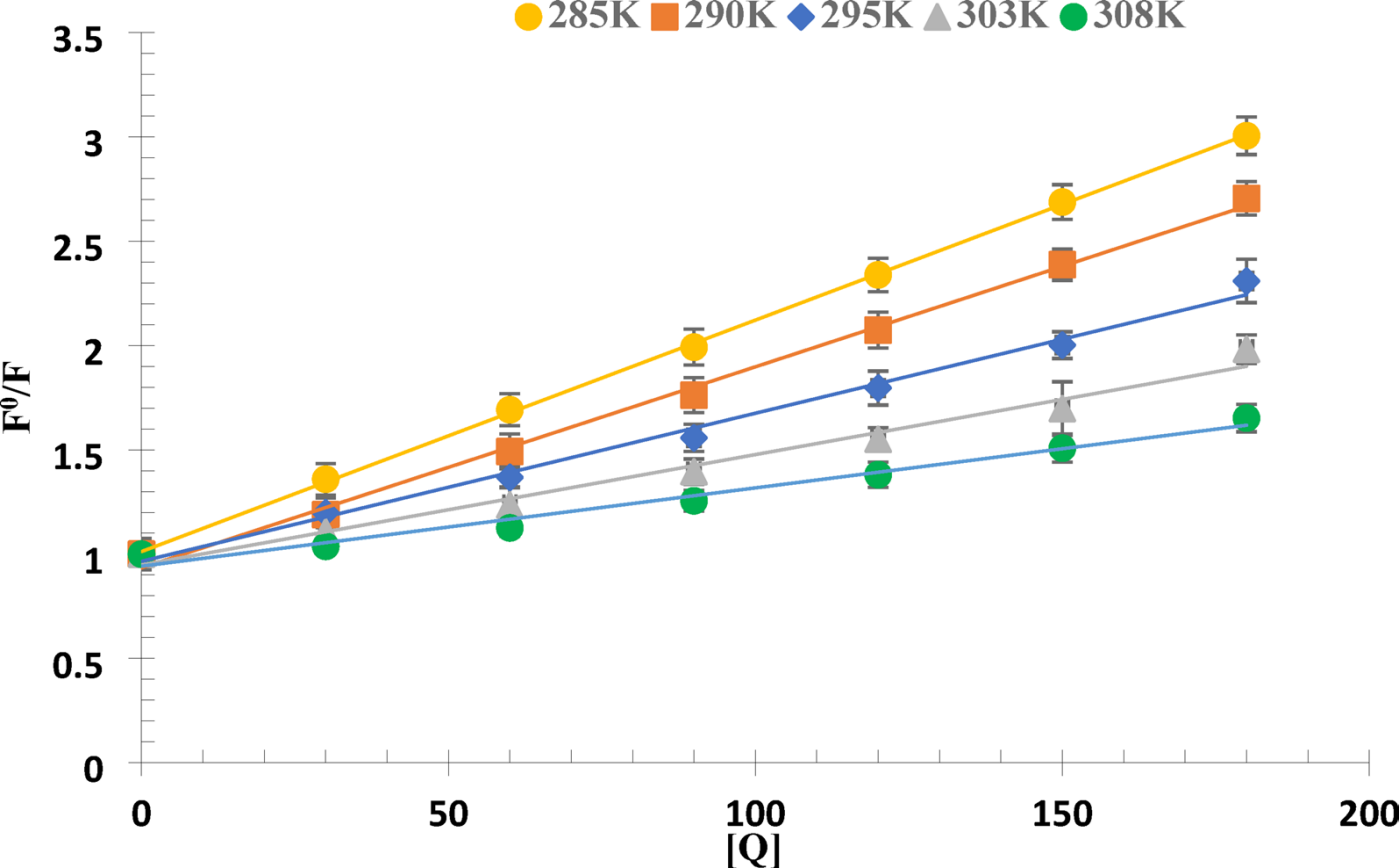
Linear Stern–Volmer plots for MOL binding to BSA at different temperatures, pH = 7.4

F^0^/F was plotted versus [Q], then the slope was calculated to obtain the values of K_sv_ as shown in the Stern–Volmer equation (Eq. 1) to assess the experimental results as shown in Fig. 3. The results showed that K_sv_ values were inversely proportion to temperatures, indicating that a static interaction between BSA and MOL mostly controlled the fluorescence quenching process. The results are listed in Table 1.

**Table 1:** Regression parameters and Stern–Volmer quenching constants for the interaction between BSA and MOL at different five temperatures

T (K)	K_sv_ (×10^3^ L/mol)	*r*	Slope±SD	Intercept ± SD	SD of residuals
285	11.1	0.9997	0.0110 ± 0.00009	1.0120 ± 0.009	0.0145
290	9.635	0.9961	0.0096 ± 0.0002	0.9340 ± 0.02	0.0427
295	7.096	0.9922	0.0070 ± 0.0002	0.9655 ± 0.03	0.0447
303	5.288	0.9805	0.0052 ± 0.0003	0.94817 ± 0.03	0.0530
308	3.80	0.9798	0.0037 ± 0.0002	0.9421 ± 0.02	0.0382

T: the absolute temperature, r: correlation coefficient

IFE-corrected fluorescence intensity values were calculated using the following formula:

$${{\text{F}}_{{\text{corrected}}}}={{\text{F}}_{{\text{observed}}}}*{\text{1}}{0^{\left( {{\text{Aex}}+{\text{Aem}}} \right)/{\text{2}}}}$$

The corrected Stern–Volmer constants (K_sv) were 7.16 × 10^3^ L/mol at 295 K compared to the uncorrected value of 7.096 × 10^3^ L/mol, showing only a 0.9% difference. This confirms that the inner filter effect is negligible under our experimental conditions.

### Stoichiometry analysis of the BSA-MOL complex

The modified Stern–Volmer equation is used to determine the stoichiometry (n) and equilibrium association constant (K_a_), which govern how small molecules interact with specific binding sites on a macromolecule (Eq. 2).

2 $$\text{log}\frac{{\text{Fo} - \text{F}}}{\text{F}}=\text{log}{\text{K}_\text{a}}+\text{n}~\text{log}\left[ \text{Q} \right]$$

where ${\text{n}}$ represents the number of binding sites and ${\text{{K}}}_{\text{{a}}}$ represents the binding constant, as calculated from the plots of log [(F_0_–F)/F] against log [Q]. The plot of the modified Stern–Volmer equation was illustrated in Fig. S2. As shown in Table 2, the slope and intercept of the plot were used to get the values of n and K_a_, respectively. The stoichiometry of the binding interaction concerning MOL and BSA was approximately 1:1, as indicated by the n value being close to unity.

**Table 2:** The thermodynamic parameters of BSA-MOL complex at various temperatures

T (K)	Log K_a_	K_a_ (×10^3^ L/mol)	*n*	*r*	Slope ± SD	Intercept ± SD	SD of residuals	ΔH (KJ.mol ^− 1^)	ΔS (J mol^− 1^K^− 1^)	ΔG (KJ mol ^− 1^)
285	4.3833	24.171	1.072	0.997	1.071 ± 0.03	4.383 ± 0.12	0.0194	-52.434	-99.63	-23.915
290	4.2414	17.434	1.077	0.998	1.077 ± 0.02	4.241 ± 0.09	0.0157			-23.546
295	4.1174	13.103	1.086	0.974	1.085 ± 0.08	4.117 ± 0.35	0.0561			-23.252
303	3.8404	6.924	1.066	0.980	1.066 ± 0.07	3.840 ± 0.30	0.0486			-22.276
308	3.6665	4.639	1.055	0.99	1.055 ± 0.05	3.666 ± 0.21	0.0336			-21.618

T: the absolute temperature, K_a_: equilibrium association constant, n: the number of binding sites, r: correlation coefficient, ΔH: enthalpy change, ΔS: entropy change, ΔG denotes the Gibbs free energy change

### Thermodynamic parameters

The intercept and slope of the plot of ln K_a_ vs. 1/T were used to determine ΔS (entropy change) and ΔH (enthalpy change), respectively in accordance with the Vant’s Hoff equation (Eq. 3), as shown in Table 2.

Furthermore, the Gibbs–Helmholtz equation determines ΔG (Eq. 4).

3 $${\text{ln}}{{\text{K}}_{\text{a}}}= - \frac{{{{\Delta {\text{H}}}}}}{{{\text{RT}}}}+\frac{{{{\Delta {\text{S}}}}}}{R}$$

4 $$\Delta {\text{G}}= - {\text{RT~ln~}}{{\text{K}}_{\text{a}}}=\Delta {\text{H}} - {\text{T}}\Delta {\text{S}}$$

where ΔG denotes the Gibbs free energy change and R denotes the universal gas constant (8.314 J mol^−1^ k^−1^) while T is the absolute temperature (K). The obtained negative values of Gibbs free energy change at the studied temperatures demonstrated the spontaneity of the interaction (Table 2). The negative values of both ΔH and ΔS indicate that the binding of MOL to BSA is primarily governed by hydrogen bonding and van der Waals interactions rather than hydrophobic forces. The thermodynamic results, was supported by docking analysis, confirming that hydrogen bonding and van der Waals contacts are the dominant driving forces in MOL–BSA complex formation. The thermodynamic results also suggest that the binding of MOL to BSA occurs naturally without external energy input. Moreover, the spontaneity of the interaction could reflect the favorable conformational stability of the MOL-BSA complex.

### Study of ultraviolet absorption

UV-Spectrophotometry is a simple and valuable technique for examining complex formation and structural changes. Any alterations in the protein’s UV absorption spectra, such as changes in intensity or λ_max_, are primarily attributed to static quenching, which occurs as a result of the ground-state complex formation. The results from this study confirmed static quenching by showing a noticeable enhancement in intensity around the 280 nm absorption band in the BSA absorption spectra in the presence of MOL, verifying static quenching, as illustrated in Fig. S3.

### Synchronous fluorescence spectroscopy

The polarity of the surrounding environment of the fluorescent amino acids in BSA is influenced by ligand interaction, and this has been studied using synchronous fluorescence spectroscopy. In this study, a scanning interval (Δλ) of 60 nm and 15 nm was set between the excitation and emission wavelengths, allowing for the observation of polarity alterations surrounding tyrosine (Tyr) and tryptophan (Trp) residues, respectively [32–34].

**Fig. 4:**
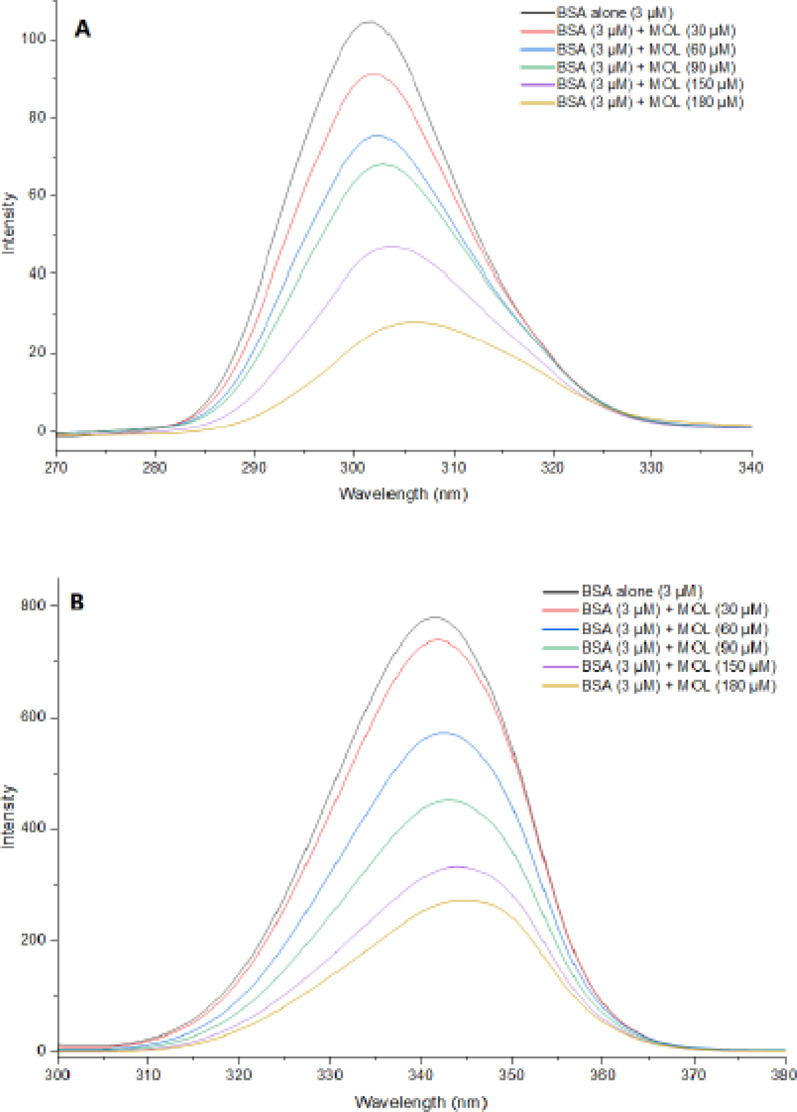
Synchronous fluorescence spectra of BSA (3 µM) with increasing concentrations of MOL (0 to 180 µM), pH = 7.4, T = 290 K. at **A** Δλ = 15 nm and at **B** Δλ = 60 nm

Spectra of BSA synchronous fluorescence were acquired for BSA solutions both with and without the addition of MOL (Fig. 4). The emission intensity of BSA exhibited significant quenching subsequent to the addition of MOL, observed at Δλ = 60 nm and Δλ = 15 nm. Furthermore, there was a slight red shift of about 2 nm in λ_max_ when Δλ was set to 15 nm (which corresponds to Tyr residues), whereas no shift was detected at Δλ = 60 nm. This suggested that MOL binding to BSA altered the microenvironment conformation around Tyr residues, a finding further supported by docking studies. The Ratios of Synchronous Fluorescence Quenching (RSFQ) were determined using Eq. 5 as follows:

5 $$RSFQ=1 - \frac{F}{{F0}}$$

where the synchronous fluorescence intensities of BSA with and without the addition of MOL are denoted by F and F^0^, respectively. As illustrated in Fig. S4, the RSFQ% values were higher for Δλ = 15 nm compared to Δλ = 60 nm. This finding confirms that MOL binds BSA molecules close to the tyrosine residues.

### Fourier-transform infrared spectroscopy (FT-IR)

FT-IR spectroscopy was utilized to examine the secondary structure of BSA in free, drug-bound forms and free drug by recording changes in spectra as shown in Fig. S5. However, slight differences were observed between these spectra. After the addition of MOL, the amide band for BSA shifted from 1610 cm^−1^ to 1636 cm^−1^. This change suggests that MOL binding causes a subtle conformational alteration in the secondary structure of BSA. The results from the UV absorption and fluorescence analysis are consistent with this observation.

### Study of the Preferential binding site of MOL on BSA

The apparent binding constants (Ka) were calculated by plotting log[(F₀–F)/F] versus log[Q] in the absence and presence of site markers Figure S6. It was observed that the Ka value for MOL binding to BSA decreased in the presence of diazepam, whereas no significant change was noted in the presence of indomethacin Table 3. The site marker displacement assay revealed a significant reduction in the binding constant of MOL in the presence of diazepam (site II probe), while indomethacin (site I probe) had little effect. These results indicate preferential binding of MOL to Sudlow’s site II, in agreement with molecular docking simulations that placed MOL within subdomain IIIA (site II).

**Table 3:** The binding constant of MOL-BSA was determined in absence and presence of site-specific markers at 295 K and pH = 7.4

Site marker	Log K_a_	*n*	*r*
Blank	4.1593	1.094	0.9817
Indomethacin	4.2494	1.056	0.997
Diazepam	3.4424	1.003	0.9955

r: correlation coefficient

### Common ions effects on the binding affinity

The impact of common ions such as Ca^2+,^ Co^2+^, and Cu^2+^ on the binding affinity of MOL to BSA were examined independently at a temperature of 295 K and 75 µM anionic concentration at pH 7.4. As shown in Table S1, the results indicated that the binding constant increased when Co was present while decreased when Ca and Cu ions were present. These findings can be used for further investigations into how different ions influence the binding affinity of MOL to BSA.

### Molecular Docking study

**Fig. 5:**
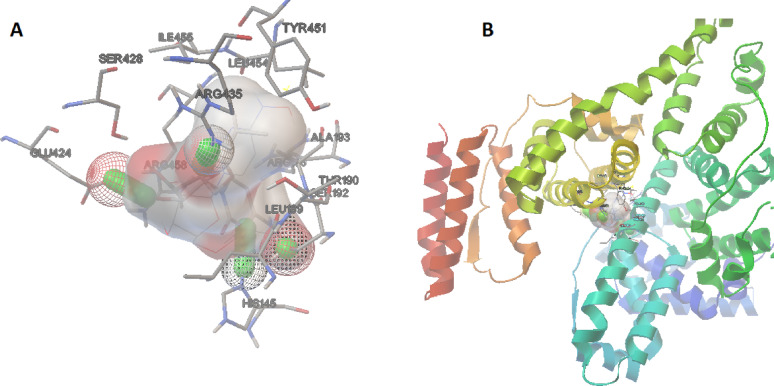
**A** Binding interactions of the best fitted docked poses of MOL in BSA subdomain III A (site II); **B** The crystal strutre of MOL-BSA complex

Molecular docking simulations were carried out using AutoDock4 to evaluate the binding mode of MOL with BSA [35, 36].The docking calculations yielded multiple conformations of the ligand, with binding energies ranging from − 5.7 to − 7.1 kcal/mol. The lowest energy pose, which had a binding energy of − 7.1 kcal/mol, was selected for further analysis. As shown in Fig. 5, the docking results revealed that MOL binds stably within a well-defined cavity located in subdomain IIIA of BSA, corresponding to Site II—a pharmacologically important site known for accommodating aromatic and anionic drug molecules such as ibuprofen and diazepam. The ligand adopts a favorable orientation, making extensive contacts within the hydrophobic cleft. Key residues involved in the binding pocket include Arg435, Arg458, Leu454, Tyr451, Glu424, Ser428, His145, Leu189, Ser192, Ala187, and Thr186. Among these, polar and charged residues such as Arg435 and Arg458 formed potential hydrogen bonds or electrostatic interactions with the carbonyl and hydroxyl groups of MOL. Additional stabilization was provided by hydrogen bonding with Ser192 and His145.

Van der Waals interactions were also significant, with Leu189, Leu454, Ala187, and Tyr451 contributing contacts that help anchor the ligand in the cavity. The binding orientation of MOL aligned well with the shape and polarity of the Site II pocket, suggesting that the interaction is both spatially and energetically favorable. The predicted binding pose and interaction profile further support the hypothesis that MOL may be efficiently transported in plasma via albumin binding, influencing its pharmacokinetics and systemic availability. Overall, AutoDock4-based docking confirmed that Site II of BSA is the preferential binding site for MOL and highlighted key stabilizing interactions, providing a valuable structural rationale for its albumin-binding potential.

Pharmacokinetic studies of MOL revealed that, following oral administration, it is rapidly absorbed and hydrolyzed by esterases to its active metabolite, *N*-hydroxycytidine (NHC), primarily during absorption and/or hepatic first-pass metabolism. As a result, no significant concentrations of the parent drug are detected systemically in plasma [37, 38]. Nevertheless, our site marker assays and molecular docking analysis consistently demonstrated that MOL binds favorably to the Site II pocket (subdomain IIIA) of serum albumin, mainly through hydrogen bonding and van der Waals interactions. These findings suggest that, although rapidly metabolized, MOL may still interact transiently with albumin prior to complete conversion, thereby influencing its initial distribution phase. Future investigations on NHC–albumin interactions will be essential to provide more physiologically relevant insights.

### Molecular dynamics simulation

To validate the conformational stability of the BSA–MOL complex and assess the consistency of binding, key MD parameters were analyzed across three independent 100 ns simulations.

**Fig. 6:**
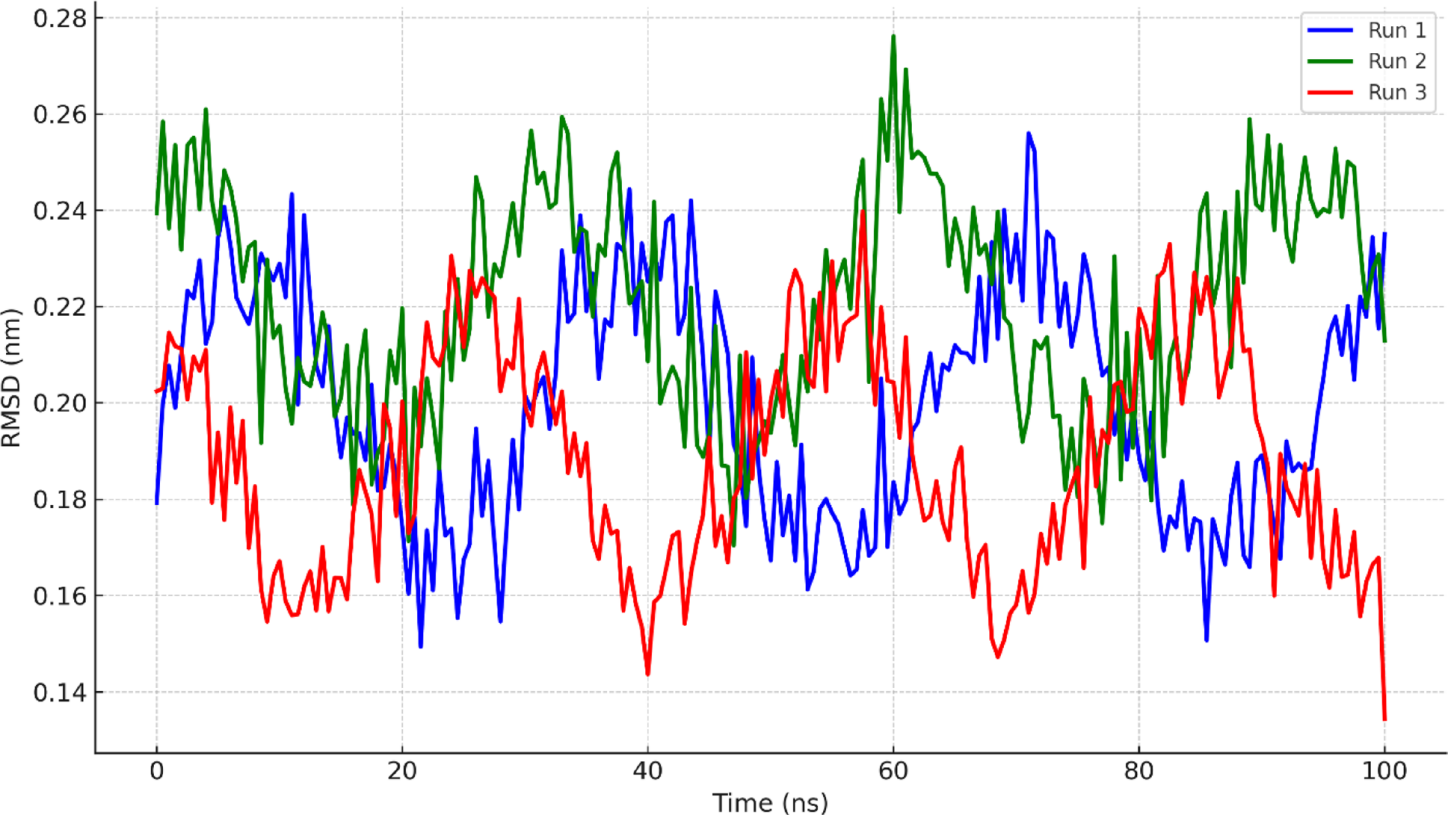
Root Mean Square Deviation (RMSD) of BSA–MOL (Triplicate)

Figure 6 shows the RMSD profiles of the BSA backbone for all three simulations. All trajectories displayed similar trends, stabilizing after approximately 10 ns and maintaining RMSD values in the range of 0.20–0.35 nm. Although minor oscillations persisted, no systematic drift was observed, suggesting that the system had reached equilibrium and was sampling a stable conformational ensemble. Such fluctuations are typical for albumins due to their flexible multi-domain architecture and do not indicate instability. The RMSD of MOL also showed stable binding within the protein’s binding pocket in all runs, with no evidence of dissociation or large displacement, further confirming robust ligand–protein interaction.

**Fig. 7:**
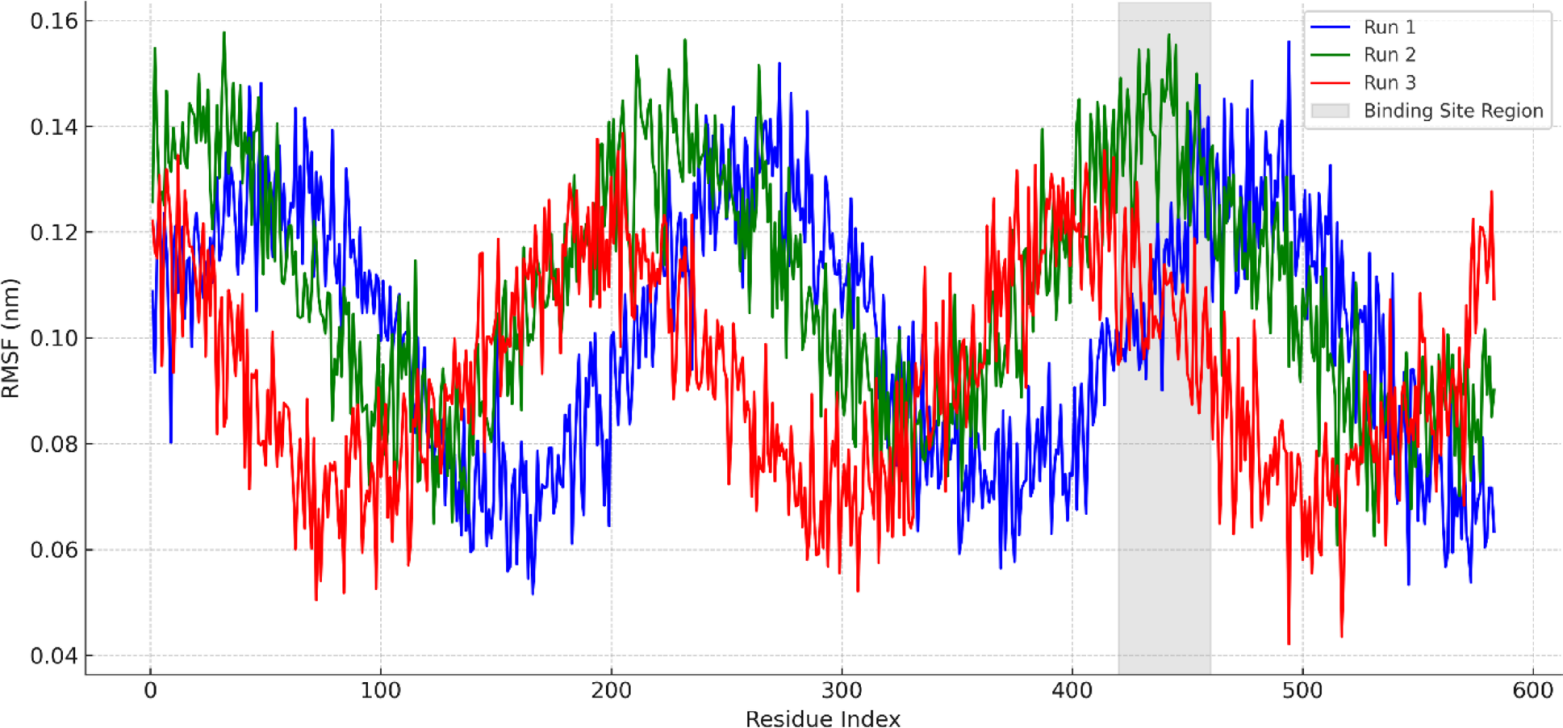
Root Mean Square Fluctuation (RMSF) of BSA residues across triplicate MD runs

Figure 7 illustrates the per-residue RMSF profiles across the three MD simulations. The majority of the residues displayed minimal fluctuations (< 0.15 nm), with particularly low flexibility observed in the ligand-binding region (residues 420–460). This region corresponds to Sudlow’s site II and includes key interaction residues such as Arg435, Tyr451, and Glu424. While some variability was seen among replicates in peripheral loop regions, the binding-site residues consistently exhibited low fluctuations, supporting reproducibility of the interaction pattern and protein flexibility behavior.

As shown in Fig. S7, the number of hydrogen bonds formed between MOL and BSA throughout the simulation ranged between 1 and 4 in all three trajectories. Despite natural fluctuations over time, the hydrogen bonding patterns remained consistent across runs, indicating the presence of both stable and transient polar interactions. Key residues involved in hydrogen bonding included Arg435, Ser192, and Glu424, which are all located within Sudlow’s site II. The consistency in hydrogen bonding frequency and distribution across the three simulations reinforces the stability and specificity of MOL binding to BSA.

The radius of gyration (Rg) values (Fig. S8) remained stable within the range of 1.9–2.2 nm, confirming that no major structural collapse or unfolding occurred during the simulations. Quantitative analysis (Table S2) shows that the backbone RMSD stabilized at 0.26 ± 0.03 nm, Rg values averaged 2.05 ± 0.04 nm, and MOL maintained 2.3 ± 0.6 hydrogen bonds with BSA across the triplicate simulations, confirming stability of the complex.

Together, the consistent RMSD, RMSF, Rg, and hydrogen bonding profiles across the three independent simulations confirm that the system had equilibrated within the 100 ns timeframe, and that the binding interactions are reproducible across replicates. While we acknowledge that extending the simulations beyond 100 ns could provide further confirmation of long-term stability, the convergence of key MD parameters across triplicates strongly validates the binding of MOL at Sudlow’s site II. These findings strongly support the docking-based predictions and suggest that MOL forms a specific and durable complex with BSA under physiological conditions.

## Conclusion

This study illustrates the interaction between MOL and BSA by employing multiple spectroscopic techniques: UV absorption spectroscopy, fluorescence spectroscopy, synchronous fluorescence spectroscopy, FT-IR, molecular docking and dynamic simulation analysis. The results show that MOL effectively quenches the intrinsic fluorescence of BSA, mostly via a static mechanism. This was further supported by UV spectroscopy results. In addition, this study has shown that MOL attaches to BSA’s Site II, spontaneously in a 1:1 stoichiometry by hydrogen bonding and van der Waals interaction, slightly altering BSA’s conformation. This study provides valuable information to better understand the molecular mechanisms of MOL, its distribution, metabolism, and therapeutic efficacy. By elucidating these aspects, this research aims to optimize the therapeutic applications of MOL and advance drug development strategies.

## Supplementary Information

Below is the link to the electronic supplementary material.

Supplementary Material 1.

## Data Availability

All data generated or analysed during this study are included in this published article and its supplementary information files.

## Abbreviations

Mol: Molnupiravir
BSA: Bovine Serum Albumin
HSA: Human Serum Albumin
Tyr: Tyrosine
Trp: Tryptophan
PDB: Protein Data Bank
Ka: Binding constant
MD: Molecular dynamics
Rg: Radius of gyration
RMSD: Root-mean-square deviation
RMSF: Root-mean-square fluctuation
