# Supplementary material for: Multi spectroscopic investigations with molecular docking and molecular dynamics simulation of the binding mechanism of molnupiravir to bovine serum albumin
Source: BMC Chem. 2025 Oct 27;19(1):286. doi: 10.1186/s13065-025-01645-5 (PMC12560410; doi:10.1186/s13065-025-01645-5)
Supplement: Supplementary file 1 — Supplementary Material 1. [file 13065_2025_1645_MOESM1_ESM.docx]

**Multi Spectroscopic Investigations with Molecular Docking and Molecular Dynamics Simulation of the Binding Mechanism of Molnupiravir to Bovine Serum Albumin**

Supplementary data:

**Fig. S1.** UV spectra of molnupiravir (80 μM) in water, pH = 7.4.

**Fig. S2.** Modified Stern-Volmer plot of log[(F_0_–F)/F] against log [Q] at different temperatures and pH 7.4.


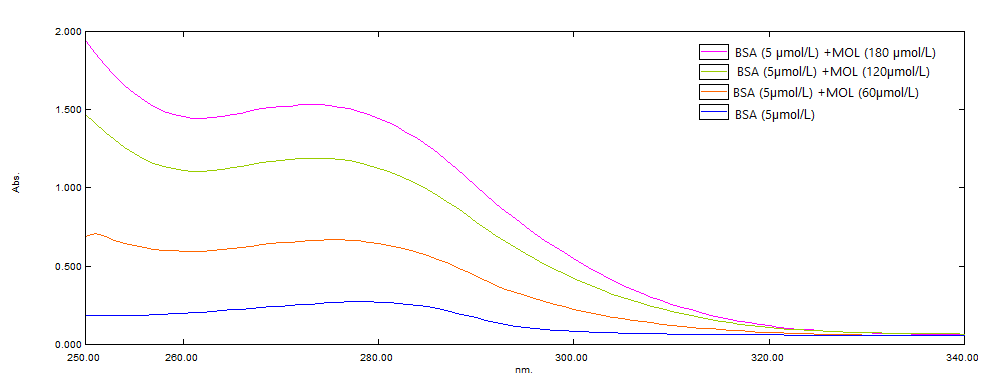


**Fig. S3** UV spectra for BSA (5 μM) alone and with increasing concentrations of MOL (0, 60, 120, and 180 μM) at 295 K, pH = 7.4.

**
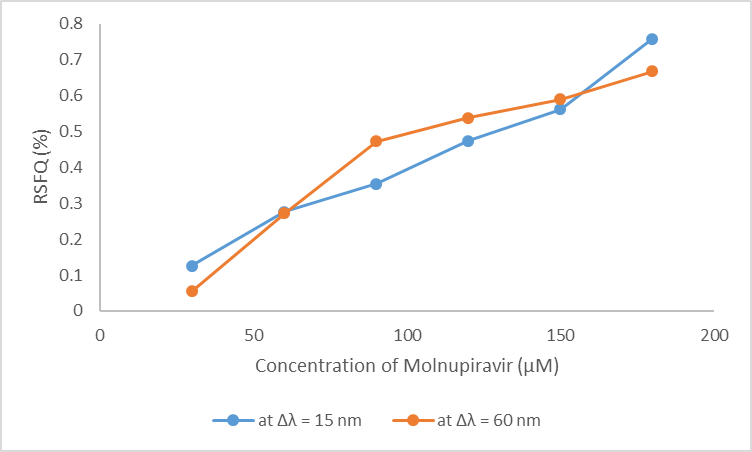
**

**Fig. S4.** Comparative analysis of MOL (30-180 μM) effect on the RSFQ% of BSA (3 μM) at pH = 7.4


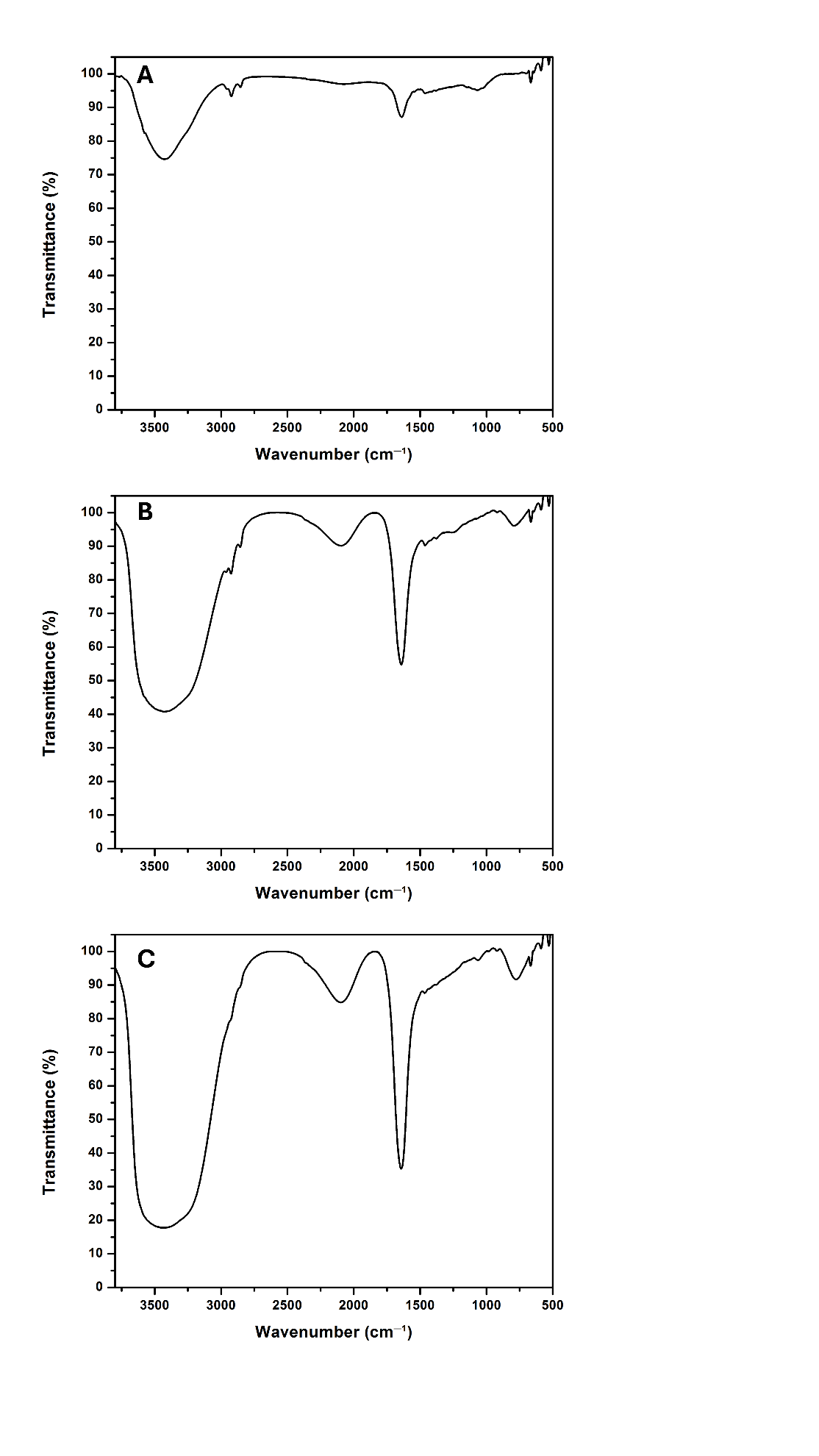


**Fig. S5.** The FT-IR spectra of (A) BSA (3 μM) in the tris-HCl buffer, (B) a mixture of BSA (3 μM) with MOL (180 µM) and (C) MOL (180 µM) in water at pH 7.4.

**Fig. S6.** Logarithmic plots of log[(F0–F)/F] against log[Q] in the presence and absence of site markers at 295 K, pH = 7.4.

**
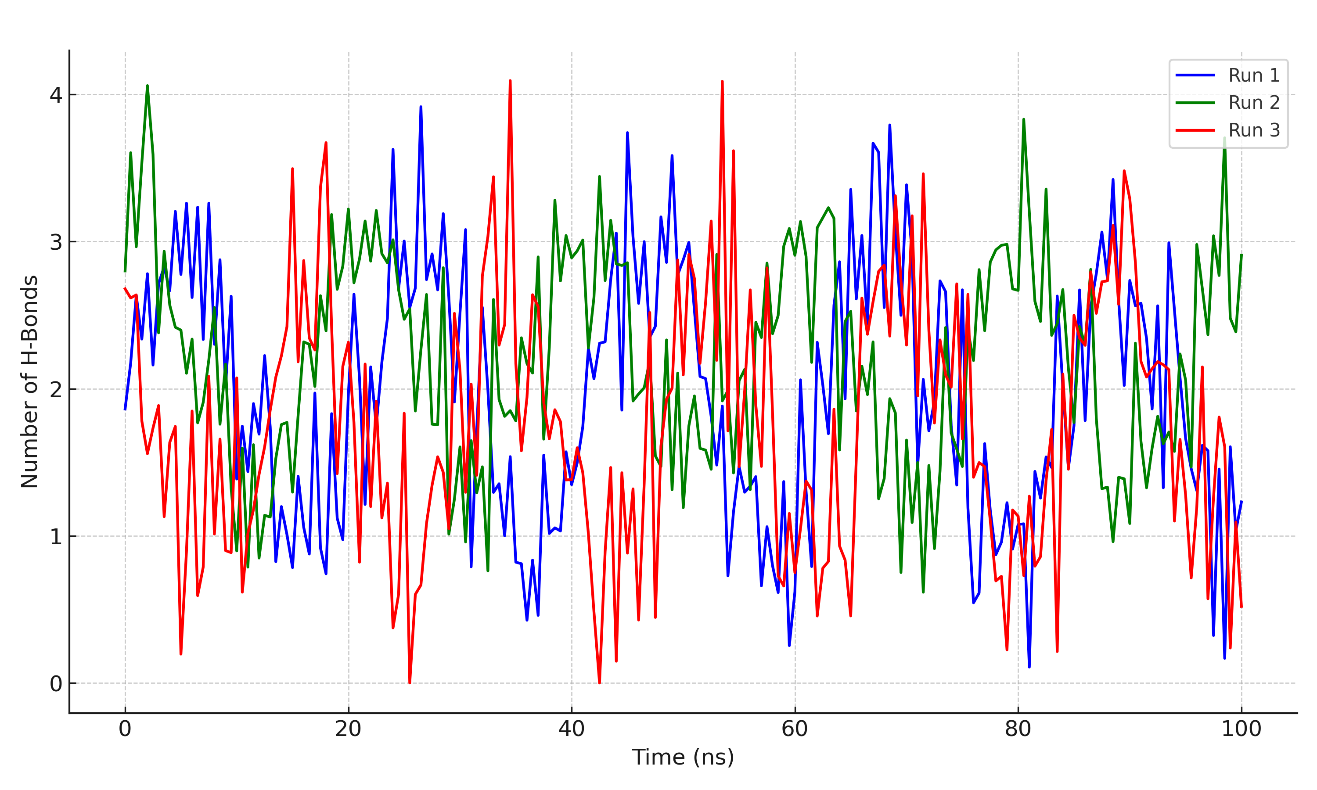
**

**Fig. S7.** Number of hydrogen bonds between MOL and BSA (Triplicate).

**
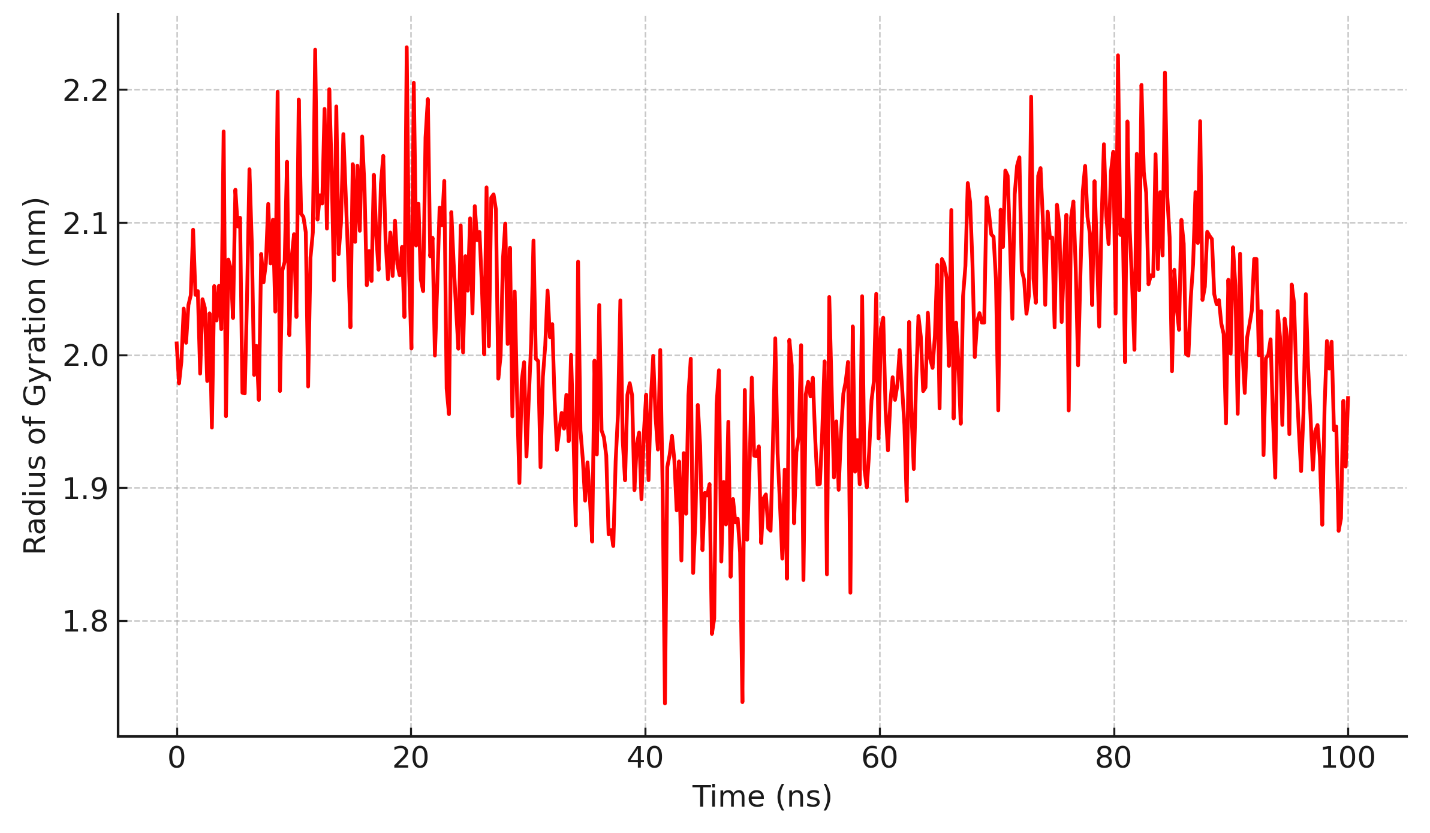
**

**Fig. S8.** Radius of gyration of BSA during MD simulation.

**Table. S1.** Binding constant of MOL with BSA in the presence of common ions at 297 K and pH 7.4.

| Ion added | Log K_a_ | K_a_’(L/mol) | K_a_’/ Ka | r |
| --- | --- | --- | --- | --- |
| No ion | 3.2151 | 1640.967 | 1 | 0.9914 |
| Ca^2+^ | 3.1716 | 1484.567 | 0.9046 | 0.993 |
| Cu^2+^ | 2.6257 | 422.376 | 0.2573 | 0.9911 |
| Co^2+^ | 3.4255 | 2663.79 | 1.6233 | 0.9958 |

K𝑎′/𝐾𝑎 is the binding constant divided by the binding constant in the absence of the ion; r is the correlation coefficient.

**Table. S2.** Mean ± SD values of key MD parameters for the BSA–Molnupiravir complex over the last 50 ns of triplicate simulations.

| Parameter | Run 1 | Run 2 | Run 3 | Mean ± SD (All Runs) |
| --- | --- | --- | --- | --- |
| Backbone RMSD (nm) | 0.25 | 0.27 | 0.26 | 0.26 ± 0.03 |
| Radius of Gyration, Rg (nm) | 2.04 | 2.06 | 2.05 | 2.05 ± 0.04 |
| Hydrogen Bonds (count) | 2.4 | 2.1 | 2.3 | 2.3 ± 0.6 |
